# Leashing heterogeneous sepsis with phenotype-informed decision support modeling

**DOI:** 10.1186/s40635-026-00948-8

**Published:** 2026-07-15

**Authors:** Joo Heung Yoon, Faraaz Ali Shah

**Affiliations:** 1 Division of Pulmonary, Allergy, Critical Care, and Sleep Medicine, University of Pittsburgh, 3459 Fifth Avenue NW 628 MUH, Pittsburgh, PA 15237 USA; 2 VA Pittsburgh Healthcare System, Pittsburgh, PA USA

**Keywords:** Precision medicine, Heterogeneity of treatment effect, Machine learning, Sepsis

Sepsis remains a syndrome defined as much by heterogeneity as by urgency. Precision medicine has identified reproducible sepsis subtypes differing in clinical outcomes and potentially treatment response [1], but the subtyping strategy may be population and purpose specific. A recent study by Hilders et al. [2] combined long short-term memory neural network modeling with a predictor network framework and reinforcement learning (RL) to yield a robust and practical longitudinal analysis of sepsis pathogenesis that could facilitate accurate and timely management decisions. This approach is not only prognostic, but also predictive in potentially guiding the optimal next best treatment decisions.

The concept of RL-guided decision support has been investigated previously and despite prior studies demonstrating potential feasibility, these earlier studies suffered from criticisms of overpromising clinical utility, patient selection, state definition design, or off-policy evaluation methods disproportionately rewarding simple decisions [3, 4]. More than anything else, the nature of complex and dynamic process of sepsis patients made the direct implementation of such policies challenging at the bedside. In the current study, the concept of phenotype-informed decision-making pivots to reflect the heterogeneity of sepsis by integrating phenotype recognition into dynamic decision-making, which is both provoking and rational.

Notably, this study, on the other hand highlights some of the ongoing challenges of translating computationally heavy data-driven modeling to real-world settings [1, 2]. First, the number of phenotypes and characterization of phenotypes are supported mainly by levels of laboratory values that might not be available in real time at the time of decision making. Second, the differences in phenotype clusters between the Amsterdam and MIMIC-IV cohorts highlight challenges when applying uniform subtyping approaches to differing populations. The authors do correlate their phenotypes with previous efforts (example: higher mortality M4 cluster in the external validation set is similar to a trajectory-based analogue of the SENECA δ phenotype), but refining phenotypes may require local recalibration. Third, like any deep learning-based research, lack of feature explanation could be an obstacle in gaining trust by clinicians at bedside. While authors attempted to overcome this limitation by using integrated gradients-based attribution maps, such approaches could be quite sensitive to baseline set up and may not reveal adequate insight of model behaviors. In addition, the counterfactual aspect of such policy was not illustrated in the current study. While this aspect of the RL model is technically hard to establish and evaluate, the understanding of counterfactual would strengthen the rationale of following RL policy, which could facilitate real life implementation [5].

Prospective studies warrant priority. The derived model would need to confront with real-world chaos: incomplete lactate draws, undocumented source control, and the recommendations that would not translate to clinical decisions. Practical questions would beget downstream hypotheses and might require more clinically informed trial attempts and human-computer interaction studies. Phenotype-informed reinforcement learning offers a genuine bridge to precision care, but only if we ensure it survives deployment to highly confounded environments where time matters most.

## Data Availability

Not applicable.
